# Spinal Cord Imaging Markers and Recovery of Volitional Leg Movement With Spinal Cord Epidural Stimulation in Individuals With Clinically Motor Complete Spinal Cord Injury

**DOI:** 10.3389/fnsys.2020.559313

**Published:** 2020-10-21

**Authors:** Enrico Rejc, Andrew C. Smith, Kenneth A. Weber, Beatrice Ugiliweneza, Robert J. Bert, Mohammadjavad Negahdar, Maxwell Boakye, Susan J. Harkema, Claudia A. Angeli

**Affiliations:** ^1^Kentucky Spinal Cord Injury Research Center, University of Louisville, Louisville, KY, United States; ^2^Department of Neurological Surgery, University of Louisville, Louisville, KY, United States; ^3^University of Colorado School of Medicine, Department of Physical Medicine and Rehabilitation, Physical Therapy Program, Aurora, CO, United States; ^4^Department of Anethesiology, Perioperative and Pain Medicine, Stanford University School of Medicine, Palo Alto, CA, United States; ^5^Department of Radiology, University of Louisville, Louisville, KY, United States; ^6^Frazier Rehabilitation Institute, University of Louisville Health, Louisville, KY, United States; ^7^Department of Bioengineering, University of Louisville, Louisville, KY, United States

**Keywords:** epidural stimulation, spinal cord injury, voluntary movement, spinal cord MRI, spinal cord lesion, spinal tracts

## Abstract

Previous studies have shown that epidural stimulation of the lumbosacral spinal cord (scES) can re-enable lower limb volitional motor control in individuals with chronic, clinically motor complete spinal cord injury (SCI). This observation entails that residual supraspinal connectivity to the lumbosacral spinal circuitry still persisted after SCI, although it was non-detectable when scES was not provided. In the present study, we aimed at exploring further the mechanisms underlying scES-promoted recovery of volitional lower limb motor control by investigating neuroimaging markers at the spinal cord lesion site *via* magnetic resonance imaging (MRI). Spinal cord MRI was collected prior to epidural stimulator implantation in 13 individuals with chronic, clinically motor complete SCI, and the spared tissue of specific regions of the spinal cord (anterior, posterior, right, left, and total cord) was assessed. After epidural stimulator implantation, and prior to any training, volitional motor control was evaluated during left and right lower limb flexion and ankle dorsiflexion attempts. The ability to generate force exertion and movement was not correlated to any neuroimaging marker. On the other hand, spared tissue of specific cord regions significantly and importantly correlated with some aspects of motor control that include activation amplitude of antagonist (negative correlation) muscles during left ankle dorsiflexion, and electromyographic coordination patterns during right lower limb flexion. The fact that amount and location of spared spinal cord tissue at the lesion site were not related to the ability to generate volitional lower limb movements may suggest that supraspinal inputs through spared spinal cord regions that differ across individuals can result in the generation of lower limb volitional motor output prior to any training when epidural stimulation is provided.

## Introduction

In an intact nervous system, supraspinal inputs to the spinal circuitry are primarily involved in volitional movement initiation and cessation, and fine motor control. Supraspinal inputs also provide a non-specific tonic drive that optimizes the spinal circuitry level of excitability to perform a motor task (i.e., walking or standing), thus capitalizing on spinal circuitry automatic properties for the control of posture and locomotion (Edgerton et al., 2004). After a severe spinal cord injury (SCI), the prevailing view is that the loss of supraspinal tonic drive to the spinal circuitry disrupts its state of excitability (Harkema, 2008; Cote et al., 2017); this fact, together with the disruption of inputs for fine motor control, leads to the inability to walk, stand and volitionally move the lower limbs. Recovery of lower limb voluntary movement in the presence of spinal cord epidural stimulation (scES) following motor complete and incomplete SCI has been demonstrated in a number of studies (Harkema et al., 2011; Angeli et al., 2014; Grahn et al., 2017; Wagner et al., 2018; Darrow et al., 2019). These findings provide evidence that the residual brain-spinal connectivity, not detectable by clinical means, can be enhanced by the scES-mediated modulation of excitability of the spinal circuitry, enabling the recovery of voluntary movement in individuals with motor paralysis.

Neuroimaging provides useful biomarkers to predict future outcomes and glean mechanistic insights following traumatic SCI (Freund et al., 2019). Magnetic resonance imaging (MRI), in particular, has been used to examine characteristics of the spinal cord and its corresponding lesion (Flanders et al., 1996; Miyanji et al., 2007; Huber et al., 2017; O’Dell et al., 2018; Vallotton et al., 2019). Both qualitative and quantitative approaches may be used to assess the severity of spinal cord damage to establish relationships with future neurological status (Miyanji et al., 2007; Talbott et al., 2015; Aarabi et al., 2017). Considering the spinal cord lesion in the axial plane, researchers are able to evaluate the extent of intramedullary cord damage in relationship to the surrounding spinal cord boundaries (Talbott et al., 2015; Smith et al., 2017). The open-source software, Spinal Cord Toolbox, allows for a standardized quantitative template-based approach to assess the integrity of white matter pathways and gray matter within the spinal cord (De Leener et al., 2017, 2018). Using this approach, spinal cord damage in the axial plane in the corresponding regions of the right and left lateral corticospinal tracts was associated with a decreased ability to generate voluntary torque in the lower extremities, in an ipsilesional manner (Smith et al., 2018). This is one example of how neuroimaging may elucidate mechanisms into recovery of motor function after SCI.

Improved mechanistic understanding of implanted spinal epidural stimulation to augment function after SCI is warranted (Rejc and Angeli, 2019), and neuroimaging is one potential approach to address this call. To the best of our knowledge, to date no studies have used quantitative MRI to investigate mechanisms involved with responsiveness to epidural stimulation after SCI.

## Materials and Methods

### Participants

Thirteen individuals (*n* = 9 males and *n* = 4 females) with chronic, clinically motor complete and sensory complete or incomplete SCI are included in this study (Table 1). The research participants signed an informed consent for lumbosacral scES implantation, stimulation, activity-based training and physiological monitoring studies, which were conducted according to the standards set by the Declaration of Helsinki, and were approved by the University of Louisville Institutional Review Board (ClinicalTrials.gov identifiers NCT02037620, NCT02339233, and NCT03364660). Prior to epidural stimulator implantation, the International Standards for Neurological Classification of Spinal Cord Injury (Burns et al., 2012) was used for classifying the injury using the ASIA (American Spinal Injury Association) Impairment Scale (AIS). The research participants were implanted with a scES unit between 3.1 and 8.6 years after SCI, and were enrolled into interventional studies focused on either the facilitation of standing and stepping, the recovery of cardiovascular function, or the recovery of cardiovascular function as well as volitional leg movements and standing (Studies 1, 2 and 3, respectively; Table 1). However, the data presented in this study were collected prior to the beginning of any intervention with scES.

**TABLE 1:** Characteristics of the research participants.

Pub ID	Age range (years)	Time between injury and surgery (years)	Injury level	AIS	Approx. lesion center	Adhesions	Decom-pression	Wallerian Deg.	Study
B30	21–25	3.2	T1	B	C6–C7	None	A, P	P	1
B23	26–30	4.2	C7	B	C5	A	A, P	A, P	1
A80	31–35	7.9	C6	A	C6	A	A, P	P	2
B21	31–35	6.9	C4	B	C5	P	P	A, P	2
A41	21–25	7.2	C4	A	C5	P	A, P	P	2
A68	31–35	3.8	C5	A	C6	A, P	A, P	P	2
A99	16–20	2.8	C4	A	C4–C5	A, P	A, P	P, min	3
B32	61–65	7.4	C4	B	C6	A	A, P	A, P	3
A101	31–35	2.4	C2	A	C3–C4	None	A	P	3
A96	26–30	3.1	C4	A	C5	A, P	A, P	A, P min	3
A110	21–25	5.8	C5	A	C7	None	A, P	A, P	3
B41	26–30	8.6	C8	B	C7	None	A	A, P	3
B47	41–45	8.2	C4	B	C4–C5	A, P	A	A, P	3

*Injury level: neurological level of the lesion by AIS (American Spinal Injury Association (ASIA) Impairment Scale; Burns et al., 2012). Approx. lesion center: vertebral level used for Spinal Cord Toolbox (De Leener et al., 2017) template registration based on the approximate lesion center. Clinical interpretation of Magnetic Resonance Imaging including presence of adhesions, type of decompression performed, and evidence for Wallerian degeneration (A, anterior; P, posterior). Each individual was enrolled in an interventional study focused on either the facilitation of standing and stepping (Study 1), the recovery of cardiovascular function (Study 2), or the recovery of cardiovascular function as well as volitional leg movements and standing (Study 3).*

### Spinal Cord MRI Collection

Prior to epidural stimulator implantation, 2-D magnetic resonance images from cervical-thoracic (C-T) and thoracic-lumbar (T-L) levels of the spinal cord in axial and sagittal views were collected. Images were obtained using a 3 Tesla system (Siemens Magnetom Skyra, Siemens Medical Solutions, Malvern, PA, United States) with Turbo Spin Echo T2-weighted pulse sequences.

Sagittal images were first obtained in two or three separate sequences to cover the spine from at least the foramen magnum to the mid lumbar or sacral regions with large field of view (FOV) images to screen patients for syringes, significant stenoses, scoliosis, levels of injury and stabilizing treatment-related surgical changes. Usually this was performed with two sequences but taller subjects required three separate sequences because of field of view limitations. Typical parameters were:

TR/TE/FA/Thick/ETL/Re_Matrix/PFOV/NSA/BW/Pixal/AQ_matrix/%samp/PE =

Upper sagittal:

3000/74/160/3 × 3.45/17/320 × 320/100%/2/600/1.125 × 1.125/320 × 240/75/442

Lower Sagittal:

3000/74/∼130/3 × 3.45/17/320 × 320/100/2/600/1.25 × 1.25/324 × 240/75/442

Where TR = repetition time, TE = echo time, ETL = echo train length, Re_Matrix = reconstruction matrix, PFOV = % phase field of view, NSA = number of signal averages, BW = bandwidth, Pixal = pixel dimensions, AQ_matrix = acquisition matrix, % samp = % sampling (or partial fourier) and PE = number of phase 3 encodes.

In a few cases, minor adjustments were made to the parameters for specific absorption rate (SAR) limitations, patient size or clinical factors. Tables with the exact values for each individual are given in Supplementary Table 1.

Additional sequential axial T2 Turbo spin echo images were obtained from the foramen magnum to the T3-4 level. In most cases, these images were obtained either with a 10% gap (standard) or no gap. Axial 5 mm images were obtained through thoracic and lumbar regions at 5 mm thickness with a 5 mm gap between images in order to reach sustainable imaging times that could be tolerated by the research participants. Example parameters are:

Cervical spine:

5190/74/160/3 × 3/26/512 × 512/100/2/610/0.35 × 0.35/256 × 179/70/260

Thoracic spine:

3690/82/121/5 × 10/28/512 × 512/100/2/610/0.35 × 0.35/256 × 179/70/252

Lumbar spine:

3280/82/121/5 × 10/28/512 × 512/100/2/610/0.35 × 0.35/256 × 179/70/252

### Spinal Cord MRI Analysis

Clinical reports were provided for each research subject prior to their inclusion in the study by a subspecialty certified neuroradiologist (RJB). In addition to screening for exclusion criteria, clinical reports included evidence (T2 hyperintensity) for Wallerian degeneration along the anterior and posterior central cord (Supplementary Figure 1), type of decompression performed, and the presence of anterior or posterior adhesions.

**FIGURE 1:**
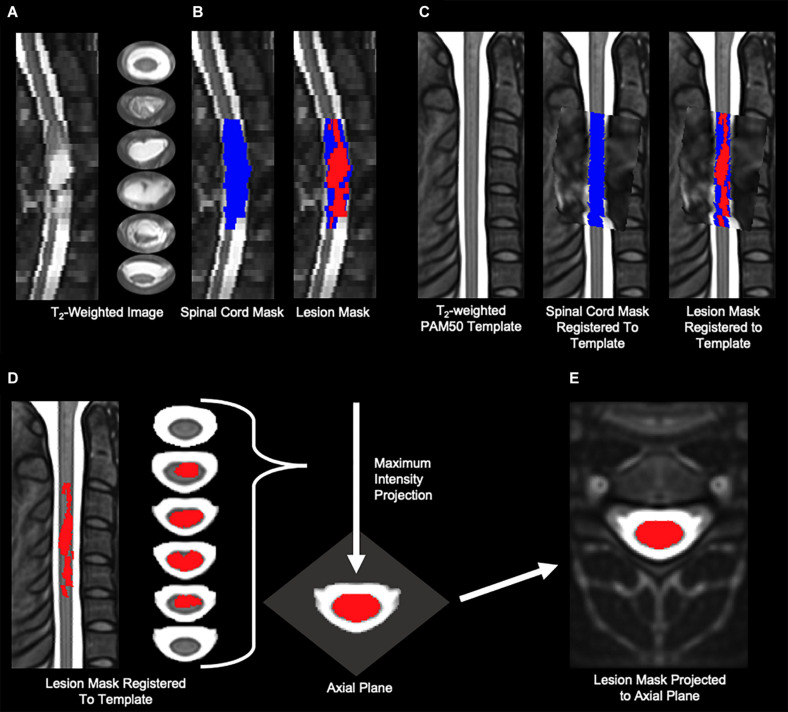
Imaging analysis methods. **(A)** Sagittal and axial slices of the T2-weighted image of a representative participant with a severe lesion. The lesion is identified as the hyperintensity in the spinal cord. **(B)** A blinded experimenter manually segmented the spinal cord (blue) and lesion (red) from the T2-weighted image. **(C)** The PAM50 T2-weighted template was then registered to the T2-weighted image using the manually draw spinal cord mask. The transformation was then applied to lesion mask to bring the lesion into template space. The spinal cord mask and lesion mask from **(B)** are shown transformed to the PAM50 spinal cord template. **(D,E)** To quantify the axial extent of the spinal cord lesion, the lesion mask in template space was then projected onto the axial plane to create one projected axial image of the composite lesion.

The open-source Spinal Cord Toolbox (Version 4.3.0) was used to calculate the amount of spared white matter (De Leener et al., 2017). SCT contains a comprehensive set of tools for the processing of multi-modal spinal cord MRI datasets including the PAM50 spinal cord standard template (resolution = 0.5 × 0.5 × 0.5 mm^3^) with a corresponding probabilistic atlas of 15 pairs (i.e., left and right side) of white matter tracts as well as functions for multi-modal registration and spatial normalization (Fonov et al., 2014; De Leener et al., 2017; Dupont et al., 2017). The individual white matter tracts within the anterior, posterior, right lateral, and left lateral spinal cord were combined to quantify the amount of spared tissue within these white matter regions (Cloney et al., 2018). Spatial normalization is the process of bringing the subject-level images into spatial agreement with a standard template. Once aligned, the subject-level images can be transformed to the template space, and the template atlases can then be used to identify corresponding anatomical regions in the subject-level images. Spatial normalization of pathological images has unique challenges (Crinion et al., 2007). In the case of spinal cord injury, the lesion appears hyperintense on T_2_-weighted images, and the spinal cord injury can also lead to spatial distortions of the lesioned and non-lesioned tissues in the cord (Talbott et al., 2019). To register the white matter atlas to the lesioned images, an experimenter blinded to the clinical history and experimental measures first manually segmented the images to generate binary spinal cord and spinal cord lesion masks. The spinal cord mask included the spinal cord lesion. The PAM50 T_2_-weighted template was then registered to the T_2_-weighted image (reference = subject) using the manually drawn spinal cord mask and PAM50 template spinal cord mask to perform the registration. The vertebral level at the center of the spinal cord lesion was used to localize the template along the superior-inferior axis. The spinal cord segmentations were then initially aligned using their center of mass, and then a series of non-linear deformations (bsplinesyn followed by columnwise) were performed to register the template spinal cord mask to the manually drawn spinal cord mask. The final registration step was performed separately for each slice (i.e., slicewise) allow for greater deformation in the warping process to account for spatial distortions in the spinal cord shape and fine-tune the registration to each slice. The utilization of the segmentation masks for spatial normalization prevented the registration from being influenced by the lesion hyperintensity. The corresponding spatial transformation was then applied to the spinal cord lesion mask to transform the lesion to template space. To quantify the spatial extent of the spinal cord lesion, the lesion mask was then projected to the axial plane, and the percentage of spared region volume (non-lesion) within the axial plane was calculated for each of the white matter regions, and these values were used for statistical analyses (Figure 1). The template registration was visually inspected at each step for quality control.

### Spinal Cord Epidural Stimulation Implant

During the scES implantation procedure, a midline bilateral laminotomy was performed typically at the L1-L2 disc space. The electrode array with 16 contacts (Medtronic Specify 5-6-5 lead) was placed into the epidural space at midline. Electrophysiological mapping was performed after initial placement to optimize the positioning of the paddle electrode based on the evoked responses recorded by surface EMG electrodes from representative lower limb muscles. After the final placement of the electrode array, the electrode lead was tunneled subcutaneously and connected to the neurostimulator.

### Voluntary Movement Experimental Procedures and Analysis

Research participants began the experimental sessions in the laboratory approximately 2–3 weeks after the surgical implantation of the spinal cord epidural stimulation unit. Spatio-temporal mapping of the spinal cord motor evoked responses was performed with the individuals relaxed in supine position (Sayenko et al., 2014; Mesbah et al., 2017; Rejc et al., 2017b). Stimulation amplitude- and frequency-response curves were used as an initial guide for the selection of stimulation parameters to facilitate voluntary leg movement (voluntary movement mapping experiments). Voluntary movement mapping took place over 1–3 days of testing. For the present study, volitional movement patterns of the right and left lower limb were assessed for ankle dorsiflexion and lower limb flexion; these motor tasks were performed in supine or semi-reclined position. In all individuals, the left and right stimulation configurations were different. Stimulation intensity was sub-motor threshold for the prime movers of the selected motor task.

Once the stimulation parameters were optimized for each individual, range of motion and force generation were assessed using either a force transducer (Kistler Holding AG, Winterthur, Switzerland) attached to a custom-built frame (Angeli et al., 2014) (*n* = 6) or a Biodex dynamometer (Biodex Inc., Shirley, NY) (*n* = 7). Electromyography (EMG) was collected bilaterally from iliopsoas, rectus femoris, vastus lateralis, medial hamstrings, tibialis anterior, and soleus. EMG data were amplified and recorded at 2,000 Hz using a hard-wired AD board and a custom-written acquisition software (LabView, National Instruments, Austin, TX), as well as band pass-filtered (30–1,000 Hz). Force and position data were also acquired with the same system and synchronized with EMG.

The background (resting) root mean square (RMS) EMG amplitude recorded prior to each volitional attempt was subtracted to the RMS EMG amplitude detected during the attempt, which was then normalized by the largest evoked potential peak-peak amplitude detected for each investigated muscle. The evoked potentials to scES were assessed with the research participants relaxed in supine position, delivering stimulation (frequency: 2 Hz; pulse width: 450 μs; electrode configuration: the three most caudal contacts (number 1, 10, 15) set as cathodes, and the three more rostral contacts (number 0, 5, 11) set as anodes) at increasing amplitude (amplitude-response curve) until either the participant requested to stop due to discomfort, or the maximum stimulator amplitude was reached. Five stimuli were delivered at each of the stimulation intensities applied; the average peak-peak amplitude for each stimulation intensity was then calculated, and the largest value recorded from each investigated muscle was considered for normalization. Quantitative information about the coordination pattern between representative muscles during volitional movement attempts (iliopsoas *vs* medial hamstrings during lower limb flexion; tibialis anterior *vs* soleus during ankle dorsiflexion) have been obtained as reported by Rejc and colleagues (Rejc et al., 2017a). Briefly, each data point of the joint probability density distribution (JPD) (Hutchison et al., 1989) represents the amplitude relationship of the EMG signals from the two muscles at a given time point. Ten percent of the largest amplitude detected during the attempt was set as threshold to define four areas of the plot representing the isolated activation of either muscle, or the co-contraction at lower or higher level of activation. The number of data points distributed in each of these four areas was finally expressed as a percentage of the total data points collected during a given attempt. Attempts with no activation detected for both muscles resulted in 100% co-contraction at low level of activation.

### Statistical Analysis

Five MRI outcomes (spared tissue of total, anterior, posterior, right, and left spinal cord) and 39 motor outcomes (number of motor tasks successfully generated, EMG amplitude of primary agonist and antagonist muscles, electromyographic coordination pattern; Supplementary Table 2) were initially considered for each research participant. We then performed the following dimensionality reduction to include only informative outcomes and important predictors. Initially, variables with zero variability were excluded because no information can be learned from them. From the remaining independent variables, we retained those that were not weakly correlated with, or were found to be important predictors, of the remaining dependent variables. The correlations were evaluated with Spearman Correlation, and the variables importance was evaluated with the Boruta Variable Importance algorithm (Kursa and Rudnicki, 2010). Any MRI outcome that was found either correlated or important for at least one motor outcome was retained. Motor variables that were not correlated with any independent variable and had no important predictor were dropped.

For count outcomes [number of motor tasks (out of four) that were successfully generated, i.e., that resulted in force exertion and movement], we used Poisson regression; for binary variables (whether force exertion and movement were detected for a given motor task), we applied the logistic regression; and for the remaining continuous variables we used the linear regression. Statistically significant MRI outcomes were combined into a multivariable regression for each motor outcome, and the adjusted estimates and *p*-values were considered for analysis. To reduce variance resulting from the relatively small sample size, we performed bagged multiple linear regressions fitted on 1,000 bootstrap copies of the data (Hastie et al., 2009). Estimates, averaged from the ensemble, were presented as the change in outcome associated with 1-unit increase in MRI measure. Data analysis was performed in SAS 9.4 (SAS Inc., Cary, NC) and R 3.6.1 (R Core Team, 2019).

## Results

**FIGURE 2:**
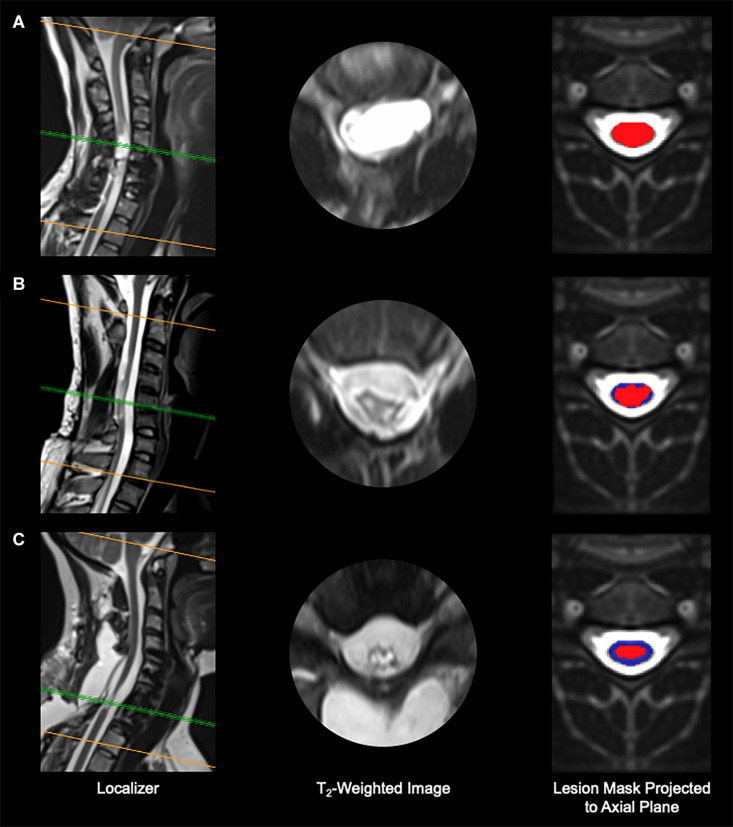
Imaging results from three representative research participants. **(A)** Participant A99, with no spared tissue of the spinal cord remaining. **(B)** Participant B21, who shows minimal spared spinal cord tissue. **(C)** Participant A110, who presents considerable spared spinal cord tissue. Red, spinal cord damage; blue, spared spinal cord tissue.

Cord MRI analysis revealed that the amount of spared tissue varied substantially across individuals, as exemplified in Figure 2. Total cord spared tissue was on average 18.6 ± 14.2%, and ranged between 0 and 42.3%.

**FIGURE 3:**
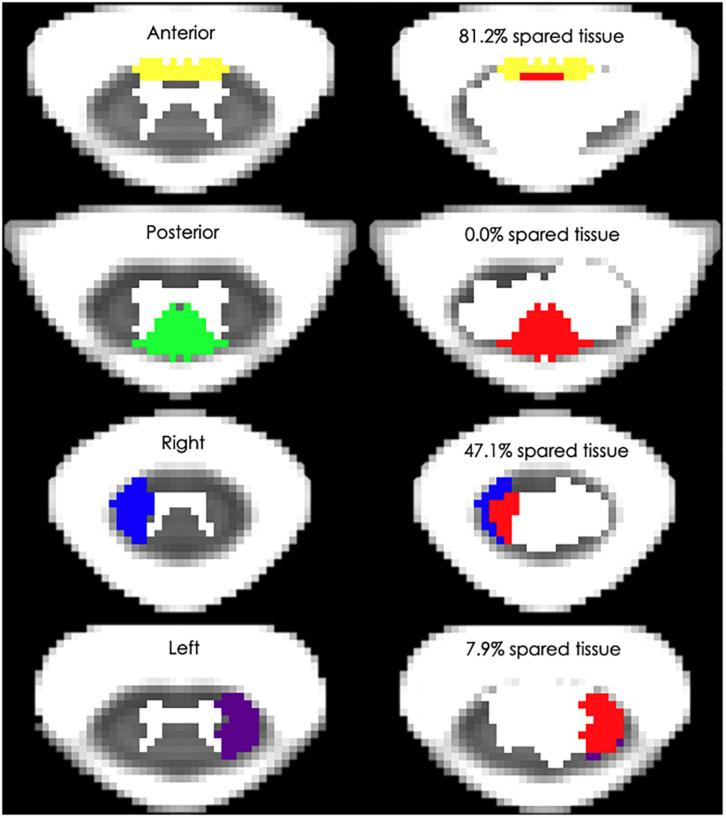
Spinal cord white matter regions and exemplary percent spared tissue. The left column depicts the four white matter regions that were used to quantify the spared tissue correlated with motor outcomes. The right column shows four representative research subjects’ spinal cord damage as well as the percentage of the corresponding white matter region that was spared. The red area indicates the lesioned tissue in each region.

Also, Figure 3 depicts the four white matter regions of interest as well as exemplary amounts of spared tissue. In particular, lateral cord spared tissue was on average 15.8 ± 16.1% (range: 0–55.9%) and 27.7 ± 24.8% (range: 0–80.0%) for the left and right side, respectively. Finally, the anterior cord spared tissue was on average 49.0 ± 36.4% (range: 0–95.8%), and the posterior 17.8 ± 17.9% (range: 0–53.2%).

On the other hand, motor responses (i.e., force generation and movement) were detected on average for 2.7 ± 1.4 motor tasks out of the four tested for each individual (range: 1–4) when scES optimized for volitional movement was applied. Conversely, no force generation and/or muscle activation was detected when research participants attempted to volitionally move the lower limb without scES.

**FIGURE 4:**
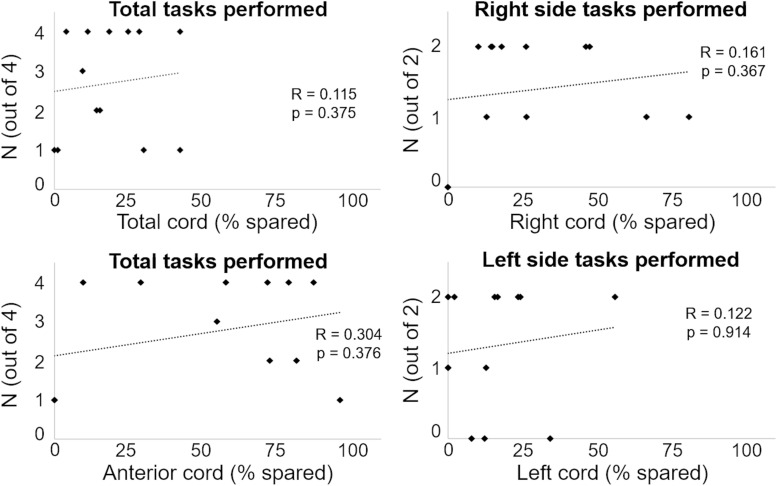
Number of motor tasks successfully performed are plotted against the amount of spared tissue of representative cord regions.

We initially tested whether the total cord spared tissue as well as the spared tissue of the four cord regions assessed in this study were correlated with the ability to generate a motor response (i.e., force exertion and movement) in the right and left side during attempts to flex the lower limb and perform ankle dorsiflexion. As exemplified in Figure 4, no significant correlations (*p*-values ranging between 0.184 and 0.985) were found between these MRI and motor outcomes.

**FIGURE 5:**
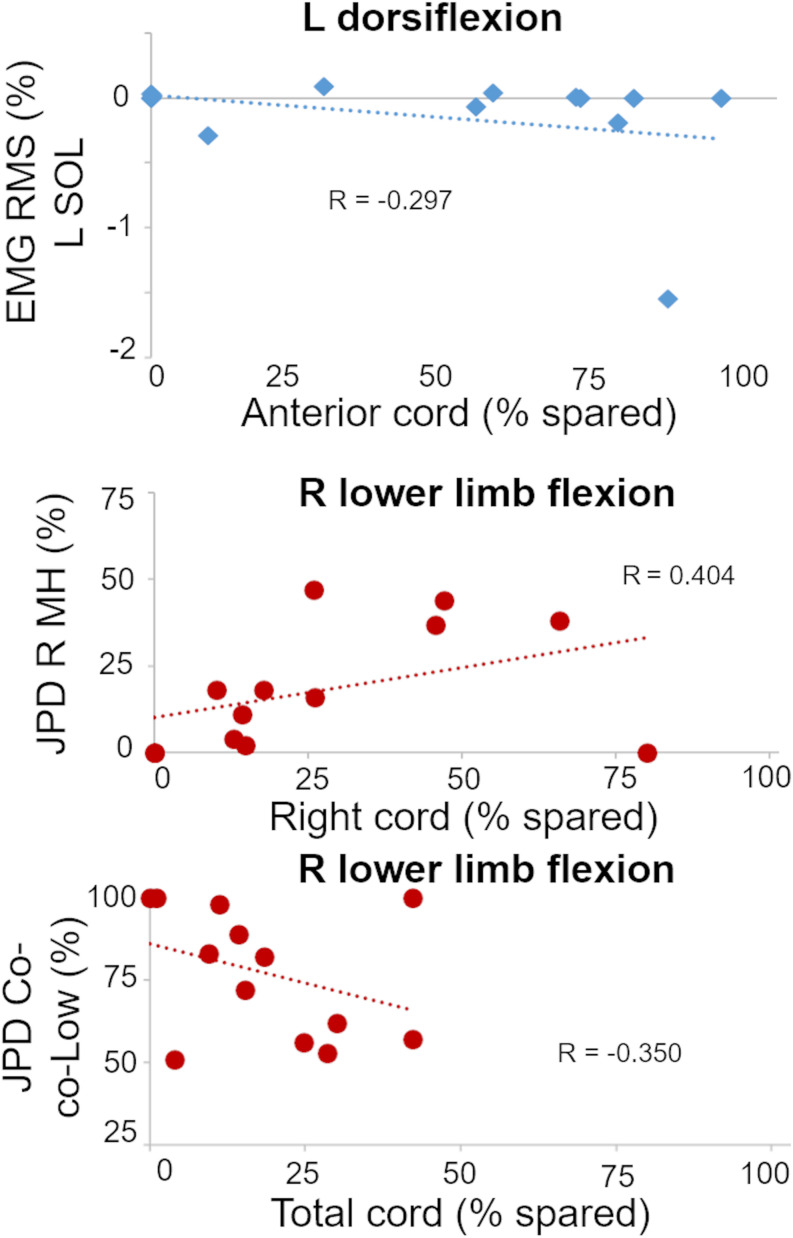
Plots of statistically significant and important correlations between amount of spared tissue of specific cord regions and muscle activation outcomes. R, right side; L, left side; RMS, root mean square; SOL, soleus; JPD R MH, amount of isolated activation of R medial hamstrings; JPD Co-co-Low, amount of co-contraction at lower level of activation between right MH and iliopsoas.

We then expanded this correlation analysis by considering the activation pattern as quantified by EMG amplitude and coordination of primary muscles involved in the attempted motor tasks, and three statistically significant and important correlations were found (Table 2). The plotting of individual data points for these three correlations between amount of spared tissue of specific cord portions and muscle activation characteristics is reported in Figure 5.

**TABLE 2:** Statistically significant and important correlations between cord MRI and motor outcomes.

Motor outcome	MRI outcome	Adjusted	
		Estimate (SE)	*p*-value
L dorsiflexion—L SOL EMG RMS	Anterior cord spared	−0.02 (0.01)	0.017
R lower limb flexion—JPD R MH	Right cord spared	0.65 (0.22)	0.015
R lower limb flexion—JPD Co-co-Low	Total cord spared	−0.987 (0.34)	0.014

*Estimated effect of the change in motor outcome associated with 1-unit increase in magnetic resonance imaging (MRI) outcome is reported, as well as the significance (p-value) of the correlation between motor and MRI outcomes. R, right side; L, left side; SOL, soleus; MH, medial hamstrings. RMS, root mean square; JPD, joint probability density distribution; JPD R MH, isolated activation of R MH; JPD Co-co-Low, co-contraction at lower level of activation between right MH and iliopsoas.*

It is worth noting that these correlations appear meaningful from a functional perspective. For example, a negative correlation between spared tissue of the anterior cord and EMG amplitude of the left soleus muscle (ankle plantarflexor; antagonist) was observed during left ankle dorsiflexion attempts (*p* = 0.017). Also, the amount of isolated activation of the right medial hamstrings (i.e., without the concurrent activation of right iliopsoas) was directly related (*p* = 0.015) with the spared tissue of the lateral right cord during right lower limb flexion attempts. Finally, for the same motor task, the amount of co-contraction at lower level of activation between right medial hamstrings and iliopsoas was inversely related (*p* = 0.014) with the total cord spared tissue.

**FIGURE 6:**
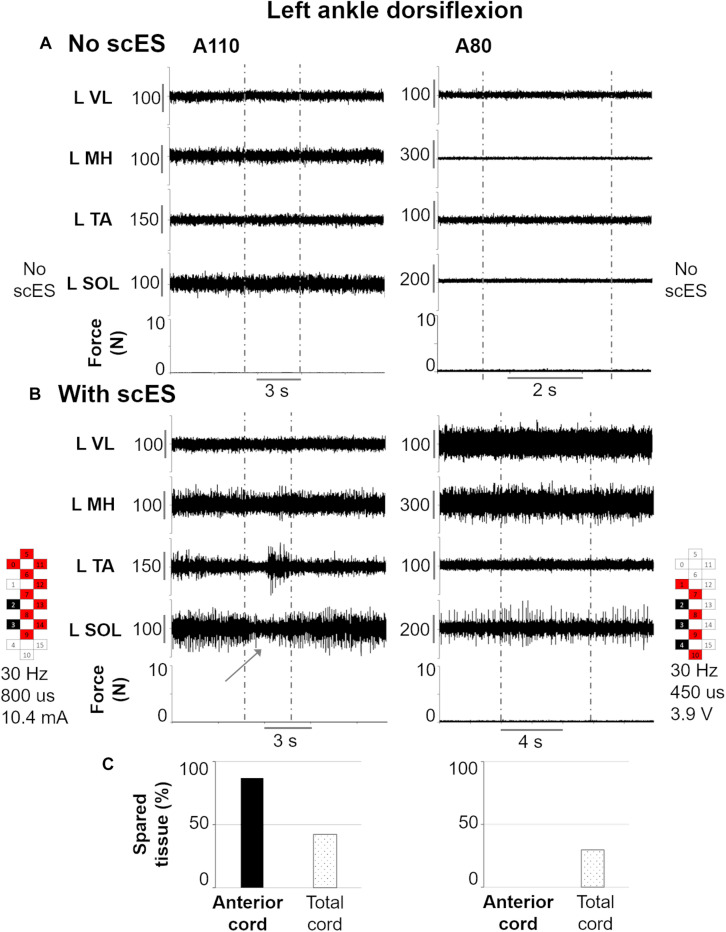
Representative EMG activity modulation and force generation during isometric ankle dorsiflexion attempts performed without **(A)** and with **(B)** spinal cord epidural stimulation (scES) by two research participants (A110 and A80). Vertical gray dotted lines: attempt duration. **(C)** Spared tissue of the anterior cord, which was found inversely correlated with EMG amplitude of L SOL (antagonist muscle) during left ankle dorsiflexion attempts, and of the total cord for the same two participants. L, left side; VL, vastus lateralis; MH, medial hamstrings; TA, tibialis anterior; SOL, soleus. The gray arrow points out the decrease in L SOL EMG activity in response to the volitional attempt. Epidural stimulation electrode configuration (cathodes in black, anodes in red, inactive in white), frequency, pulse width and intensity are reported.

Figure 6 exemplifies the effects of scES during attempts to perform left ankle dorsiflexion in individuals that showed different amount of anterior cord spared tissue, which was found correlated with the activation amplitude of a key antagonist muscle (soleus, SOL) for this motor task. As expected, no movement or EMG modulation was observed in response to volitional attempts without scES (Figure 6A). Also, no force exertion was detected when scES was applied (Figure 6B). However, in this condition, the individual with greater amount of anterior cord spared tissue (A110) demonstrated a decrement in SOL EMG activity when attempting to dorsiflex the ankle joint, while a concurrent EMG burst of the tibialis anterior muscle (agonist) was also detected. Conversely, no EMG activity modulation was observed during the same motor task in the other individual (A80), who was diagnosed with no anterior cord spared tissue, while still demonstrating 30% of total cord spared.

**FIGURE 7:**
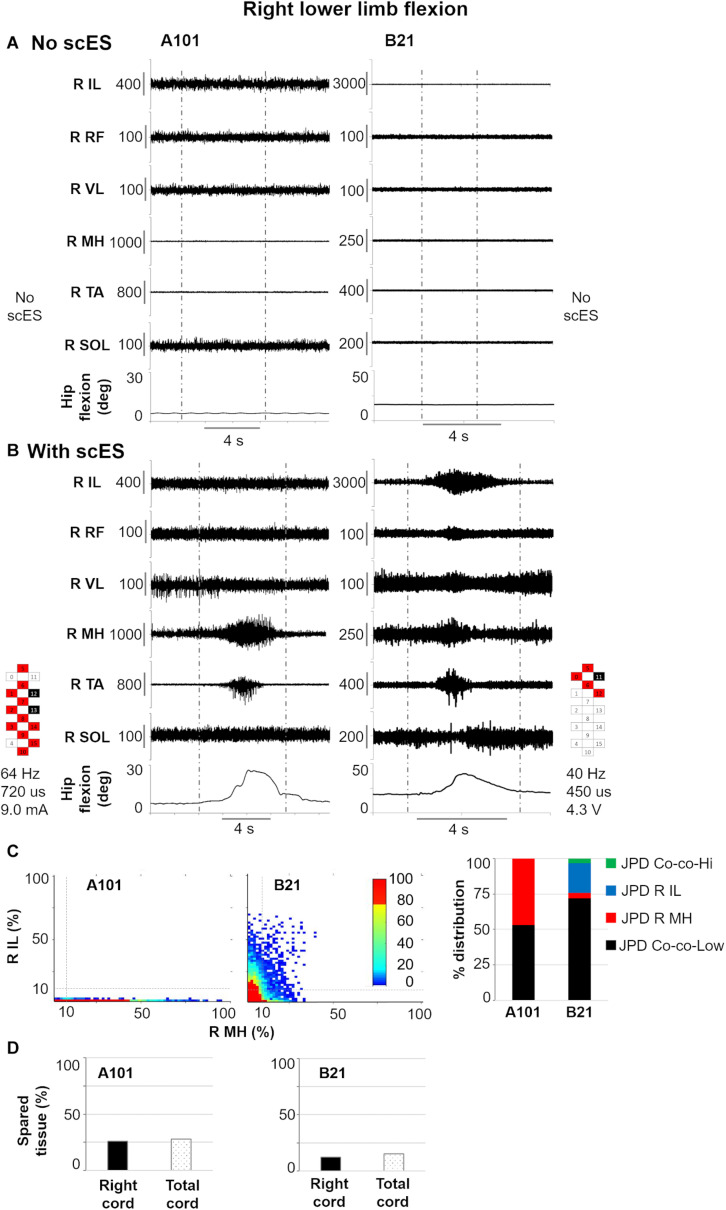
Representative EMG activity modulation and hip joint movement during right lower limb flexion attempts performed without **(A)** and with **(B)** spinal cord epidural stimulation (scES) by two research participants (A101 and B21). Vertical gray dotted lines: attempt duration. **(C)** Probability density distribution (JPD) of normalized EMG amplitudes between right (R) iliopsoas (IL) and medial hamstrings (MH) calculated during the volitional attempts, and related data points distribution in each of the four identified areas [co-contraction at lower (JPD Co-co-Low) or higher (JPD Co-co-Hi) level of activation; isolated activation of R IL (JPD R IL) or R MH (JPD R MH)]. **(D)** Spared tissue of the right and total cord, which were found correlated with JPD R MH and JPD Co-co-Low, respectively, during right lower limb flexion attempts. RF, rectus femoris; VL, vastus lateralis; TA, tibialis anterior; SOL, soleus. Epidural stimulation electrode configuration (cathodes in black, anodes in red, inactive in white), frequency, pulse width and intensity are reported.

Other two significant relationships between EMG activity modulation and cord spared tissue are exemplified in Figure 7, which focuses on right lower limb flexion. As expected, no movement or EMG modulation was observed in response to volitional attempts without scES (Figure 7A). On the other hand, when scES was applied, both research participants were able to volitionally flex the lower limb (Figure 7B). However, greater amount of isolated activation of right medial hamstrings as well as lower amount of co-contraction at low level of activation between right iliopsoas and medial hamstrings were found in participant A101, who showed greater right cord spared tissue (26 vs. 12%) as well as total cord spared tissue (28 vs. 15%) as compared to participant B21.

## Discussion

A traumatic SCI diagnosed as chronic, clinically motor complete can manifest different neurophysiological and anatomical (Figures 2, 3) profiles. For example, it is not uncommon to detect activation of paralyzed leg muscles in response to volitional movement attempts, with or without concurrent “reinforcement maneuvers” resulting from volitional activation of muscles above the level of injury (i.e., motor discomplete SCI) (Dimitrijevic et al., 1984; Dimitrijevic, 1988; McKay et al., 2004). Clinically motor complete SCI can also be characterized by the lack of these neurophysiological responses, while still presenting limited spared neural connectivity across the lesion; in fact, most of the clinically complete SCI are not anatomically complete (Kakulas, 2004). Interestingly, we have previously observed that 7 individuals with this profile of chronic SCI (clinically motor complete, without the ability to generate EMG activity in the paralyzed muscles during volitional attempts with and without reinforcement maneuvers) were able to volitionally generate meaningful leg muscles activation and movements in the presence of subthreshold levels of scES before the occurrence of any activity-based training with scES (Angeli et al., 2014, 2018).

In the present study, none of the research participants were able to modulate the baseline EMG activity of lower limb muscles by volitionally attempting to generate lower limb flexion or ankle dorsiflexion when scES was not present (Figures 6A, 7A). Conversely, all individuals were able to generate meaningful volitional motor output (generation of force and movement, and/or activation of primary muscles involved in the movement attempt) when scES was present (i.e., Figures 6B, 7B). The regaining of volitional lower limb muscle activation and movement has been interpreted as scES modulated the excitability of lumbosacral spinal circuitry, thus allowing the supraspinal input to travel across small and dormant spared fibers, engaging the appropriate spinal networks to generate the desired motor pattern. This perspective that low-intensity scES can “amplify” the residual supraspinal input is supported by experimental findings in a rat model, showing that the amplitude of motor potentials evoked from cortical stimulation was substantially increased in the presence of timed sub-motor threshold scES (Mishra et al., 2017). However, the volitional leg movement ability assessed in this study varied substantially across participants; in fact, volitional movement and force exertion was observed on average for 2.7 ± 1.4 motor tasks out of the 4 tested, ranging between 1 and 4 tasks for each individual. This inter-subject variability may suggest that individual-specific characteristics may play an important role in the extent of motor function recovery promoted by scES (Dietz, 1997; Hiersemenzel et al., 2000; Beauparlant et al., 2013). Herein, we have made an effort to explore this topic further, attempting to understand whether the amount of spared tissue of specific spinal cord regions is related to the recovery of lower limb volitional movement with scES.

In the present study we found that amount and location of spared spinal cord tissue at the lesion site were unrelated to the ability to generate volitional lower limb movements with scES (Figure 4). Residual descending inputs often undergo neuroplastic sprouting into intact pathways below the level of injury in order to communicate with their distal targets (Courtine et al., 2008; Rosenzweig et al., 2010; Asboth et al., 2018). For example, reorganization of the corticospinal system to propriospinal and brainstem pathways have been indicated in motor recovery (Courtine et al., 2008; Filli and Schwab, 2015; Asboth et al., 2018). Spinal cord sprouting of individual-specific spared tissue may have occurred in our participants, resulting in volitional lower limb motor recovery when scES is applied. Supraspinal reorganization has also been demonstrated to play a role in recovery of motor function after SCI (Winchester et al., 2005), and this is also a research avenue of interest in the context of scES. Furthermore, it is also important to acknowledge that factors other than the spared neural tissue across the lesion site play a critical role for the scES-promoted motor function recovery. For example, the appropriate selection of scES parameters, which are individual- and task-specific, crucially affects the extent and characteristics of motor recovery (Gad et al., 2013; Rejc et al., 2015; Mesbah et al., 2019; Rejc and Angeli, 2019). Additionally, recent findings also suggest that the position of the electrode array with respect to the conus tip as well as the amount of lumbosacral enlargement coverage by the electrode array concur to determine volitional lower limb movement ability in this population prior to any activity-based intervention (Ball et al., 2019).

On the other hand, we found MRI spinal cord measures related to some aspects of motor control. In primates, approximately 2% of corticospinal fibers descend ipsilaterally in the anterior corticospinal tract (Rosenzweig et al., 2009). Following SCI, lateral corticospinal tract fibers show capacity to project to this anterior column region (Steward et al., 2008), and this neuronal sprouting has been associated with functional recovery (Weidner et al., 2001). For our study, anterior spinal cord spared tissue was related to less antagonist muscle activation (i.e., lower soleus EMG amplitude) during volitional dorsiflexion attempts of the left ankle (Table 2 and Figure 6), suggesting that residual descending inputs can access inhibitory pathways after severe SCI (McKay et al., 2004) when scES is applied for facilitating volitional leg movement. Neuroplastic changes of the lateral and anterior corticospinal systems may be involved in this finding; however, it is important to consider that both spinal cord MRI and volitional motor function were assessed prior to any training with scES. Hence, these potential neural adaptations would be the result of spontaneous plasticity following SCI, and/or would have been facilitated by standard rehabilitative approaches performed prior to epidural stimulation implantation. We also found the amount of isolated activation of the right medial hamstrings during lower limb flexion attempts to be significantly related to spared tissue of the ipsilateral spinal cord (Table 2 and Figures 5, 7). This result is in alignment with a previous study involving a cohort with less severe SCIs (motor incomplete), where integrity of the lateral corticospinal tract was related to ipsilateral lower limb torque production (Smith et al., 2018). For the same motor task, the amount of co-contraction at lower level of activation between right medial hamstrings and iliopsoas was inversely related with total spared tissue, suggesting that higher level of activation of either muscle, or both muscles concurrently, may be facilitated by overall greater spinal cord spared tissue. Interestingly, spared tissue in the posterior cord was not related to any motor outcome. This is in line with the anatomical perspective that the posterior column conveys light touch and non-noxious sensory afferent information. One future direction of this research is also to investigate the role of spared posterior cord tissue in sensory recovery when sES is applied. Overall, it is worth noting that spared spinal cord tissue characteristics at the lesion site were conceivably related to residual descending inputs that affected some aspects of motor coordination (i.e., inhibition of antagonist muscles; muscle activation synergies) rather than directly and solely affecting the level of excitability of motor pools primarily involved in the attempted motor task. This observation seems interesting in the context of motor recovery after SCI, as previous evidence suggests that improving intra- and inter-limb coordination of motor pools is a primary adaptation in the process of re-learning to perform a motor task (Edgerton and Roy, 2009). For example, the progression from inability to achieve volitional muscle activation, to the co-activation of agonist, antagonist and distant muscles, to a more refined, task-specific activation pattern and movement generation has been observed following long-term activity based training with scES (Rejc et al., 2017a) as well as in individuals with incomplete SCI (McKay et al., 2011), suggesting the occurrence of neural reorganization involving inhibitory and excitatory interneurons. Locomotor training, which can promote significant motor recovery after incomplete SCI, also results in significant activity-dependent plasticity involving inhibitory interneurons (Knikou et al., 2015).

### Limits of the Study

The imaging approach in the present study has been recently applied to an incomplete spinal cord injury cohort, with lateral corticospinal tract regions correlating with their corresponding lower extremity motor output, and a high level of reliability (Smith et al., 2018). Also, similar methods using the Spinal Cord Toolbox have been applied to a cohort of acute myelitis (McCoy et al., 2017). However, an intrinsic limitation of these imaging approaches is that they provide an *estimate* of spinal cord damage location based on MRI. MRI can provide high-resolution volumetric information with excellent visualization of the lesion hyperintensity. However, the spinal cord lesion itself distorts the spinal cord dimensions in the axial plane, and the majority of the participants had undergone spinal cord fusion with implanted metallic surgical hardware, which can also cause imaging artifacts (Hargreaves et al., 2011). Importantly, none of the participants of the present study had visible imaging artifacts extending into the spinal cord; nevertheless, cord distortions could influence the accuracy of the white matter atlas registration and the localization of the lesion to the respective white matter tracts (Talbott et al., 2019). Also, for this study we used available clinical scans which had moderate resolution yet may not be able to detect minute amounts of spared tissue, as we observed that one individual with estimated 0% spared tissue achieved one volitional movement out of the four attempted tasks. Future studies applying image acquisition methods with higher spatial resolution (<1 mm^3^) and reduced susceptibility to metal as well as improved image analysis methods will further enhance our ability to localize and quantify the lesion after SCI with quantitative spinal cord MRI (Alley et al., 2018; Shi et al., 2019).

Additionally, the epidural stimulation implants utilized in this study are not 3T-MRI compatible. This does not allow the assessment of the same research participants longitudinally in order to investigate the effects of activity-based training with scES on the neural tissue at the lesion site, possibly elucidating which neural adaptations might be related to the training-induced improvements of lower limb volitional motor control.

As discussed above, the selection of stimulation parameters plays a critical role in characterizing the motor pattern promoted by scES. Each research participant underwent between 1 and 3 experimental sessions aimed at defining appropriate stimulation parameters prior to assessing volitional leg movement ability. While this procedure followed consistent guidelines and was performed by two operators with similar and extensive expertise in the field, it is possible that the level of optimization of scES parameters might have been slightly differed across participants.

In conclusion, the amount and location of spared spinal cord tissue at the lesion site were not related to the ability to generate epidural stimulation-promoted volitional joint movement and/or force exertion during attempts to flex the lower limb and dorsiflex the ankle in individuals with chronic motor complete SCI prior to any activity-based training. On the other hand, spared tissue of specific cord regions were related to some aspects of motor control. These findings may suggest that supraspinal inputs through spared spinal cord regions that differ across individuals can result in the generation of lower limb volitional movements prior to any training when epidural stimulation is provided. Future studies aimed at detailing the spared tissue of specific white matter pathways using higher resolution MRI, and pairing imaging and neurophysiology markers appear important to provide further mechanistic insights on the recovery of volitional leg movements as well as other motor functions (i.e., standing and stepping) promoted by epidural stimulation after severe spinal cord injury.

## Data Availability Statement

The raw data supporting the conclusions of this article will be made available by the authors, without undue reservation, to any qualified researcher.

## Ethics Statement

The studies involving human participants were reviewed and approved by the Institutional Review Board at the University of Louisville. The patients/participants provided their written informed consent to participate in this study.

## Author Contributions

ER and AS contributed to the study conception. ER, AS, SH, and CA contributed to the study design. RB and MN contributed to spinal cord MRI collection. MB performed epidural stimulation implantation. CA, SH, and ER contributed to voluntary movement data collection and analysis. AS, KW, and RB contributed to spinal cord MRI analysis. BU contributed to the statistical analysis. ER, AS, KW, and RB created the figures. ER, AS, and KW wrote the first draft of the manuscript. All authors contributed to the interpretation of results, revised the manuscript and approved its final version.

## Conflict of Interest

The authors declare that the research was conducted in the absence of any commercial or financial relationships that could be construed as a potential conflict of interest.

## Supplementary Material

Supplementary Table 1 Spinal cord MRI collection parameters utilized for each research participant.

Supplementary Figure 1 Wallerian degneration. **(A,C)** Sagittal and axial T2 Turbo spin-echo images through the spinal cord at the C2 level (yellow line), above a long segment injury from C4 to C6. The arrow points to wedge-shaped T2 hyperintensity at the dorsal columns. The signal extends linearly slightly anterior to the center of the cord. **(B,D)**. Sagittal and axial T2 Turbo spin-echo images through the spinal cord at the C4-5 level in a different subject, above a T1–T2 injury. The dorsal column T2 hyperintensity is less wedge-shaped in this subject and linear T2 hyperintensity extends to the anterior surface of the cord.

Supplementary Table 2 Motor outcomes considered for analysis.
