# Supplementary figures and images for: Spinal Cord Imaging Markers and Recovery of Volitional Leg Movement With Spinal Cord Epidural Stimulation in Individuals With Clinically Motor Complete Spinal Cord Injury
Source: Front Syst Neurosci. 2020 Oct 21;14:559313. doi: 10.3389/fnsys.2020.559313 (PMC7654217; doi:10.3389/fnsys.2020.559313)

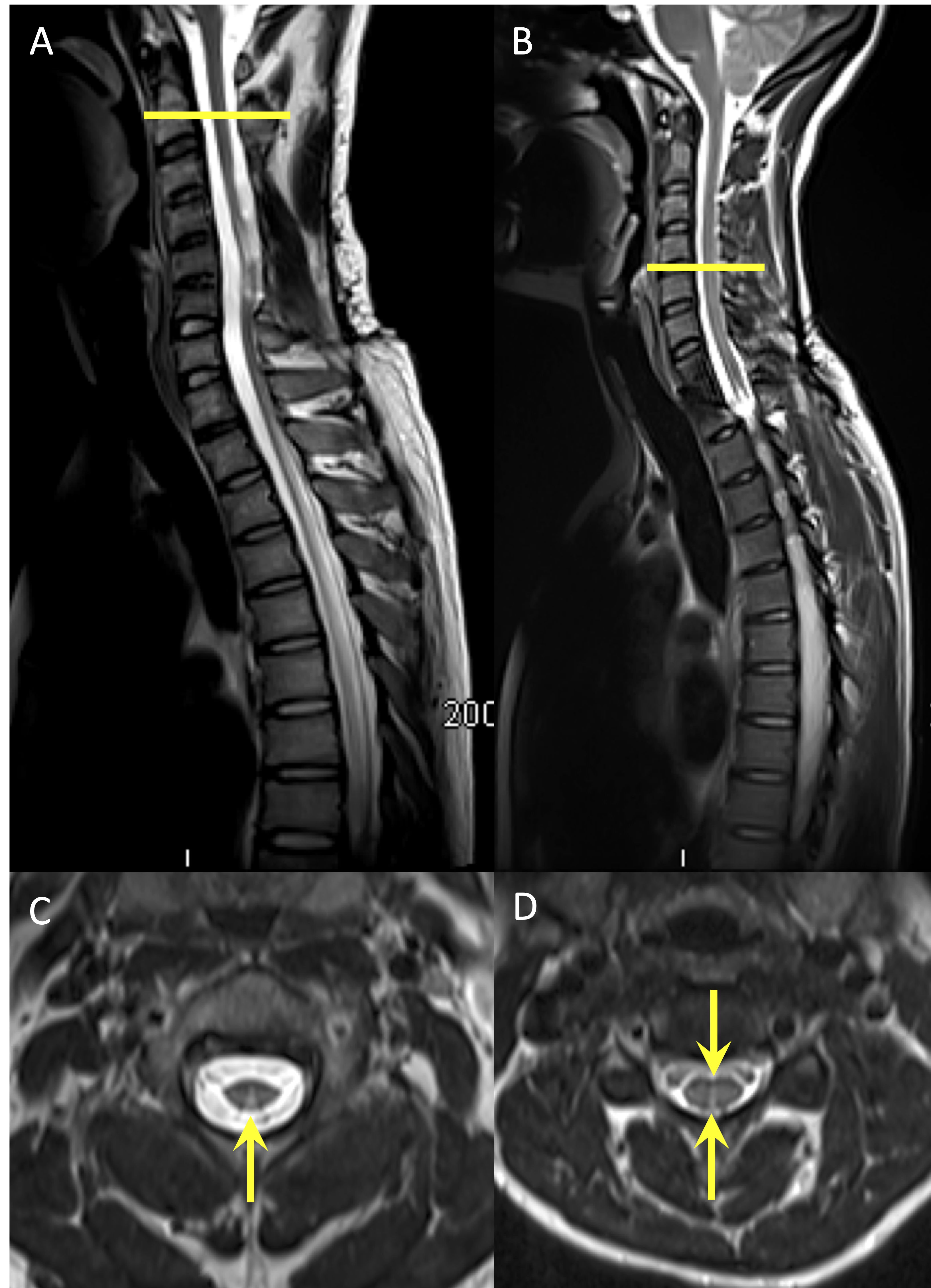

Supplement: Supplementary Figure 1 — Wallerian degneration. (A,C) Sagittal and axial T2 Turbo spin-echo images through the spinal cord at the C2 level (yellow line), above a long segment injury from C4 to C6. The arrow points to wedge-shaped T2 hyperintensity at the dorsal columns. The signal extends linearly slightly anterior to the center of the cord. (B,D). Sagittal and axial T2 Turbo spin-echo images through the spinal cord at the C4-5 level in a different subject, above a T1–T2 injury. The dorsal column T2 hyperintensity is less wedge-shaped in this subject and linear T2 hyperintensity extends to the anterior surface of the cord. [file Image_1.TIFF]
