# Supplementary material for: Spinal Cord Imaging Markers and Recovery of Volitional Leg Movement With Spinal Cord Epidural Stimulation in Individuals With Clinically Motor Complete Spinal Cord Injury
Source: Front Syst Neurosci. 2020 Oct 21;14:559313. doi: 10.3389/fnsys.2020.559313 (PMC7654217; doi:10.3389/fnsys.2020.559313)
Supplement: Supplementary Table 2 — Motor outcomes considered for analysis. [file Table_2.DOCX]

**Supplemental Table 1.** Motor tasks and related outcomes.

| **R lower limb  flexion** |  | **R ankle dorsiflexion** |  | **L lower limb flexion** |  | **L ankle  dorsiflexion** |
| --- | --- | --- | --- | --- | --- | --- |
| Total motor task performed (N *out of 4*) | | | | | | |
| R side task performed (N *out of 2*) | | |  | R side task performed (N *out of 2*) | | |
| Task performed (*y/n*) |  | Task performed (*y/n*) |  | Task performed (*y/n*) |  | Task performed (*y/n*) |
| R IL EMG RMS |  | R TA EMG RMS |  | L IL EMG RMS |  | L TA EMG RMS |
| R RF EMG RMS |  | R SOL EMG RMS |  | L RF EMG RMS |  | L SOL EMG RMS |
| R MH EMG RMS |  | JPD Co-co-Low |  | L MH EMG RMS |  | JPD Co-co-Low |
| R TA EMG RMS |  | JPD R TA |  | L TA EMG RMS |  | JPD L TA |
| R FLEXORS EMG RMS |  | JPD R SOL |  | L FLEXORS EMG RMS |  | JPD L SOL |
| JPD Co-co-Low |  | JPD Co-co-Hi |  | JPD Co-co-Low |  | JPD Co-co-Hi |
| JPD R IL |  |  |  | JPD L IL |  |  |
| JPD R MH |  |  |  | JPD L MH |  |  |
| JPD Co-co-Hi |  |  |  | JPD Co-co-Hi |  |  |

R: right side; L: left side; IL: iliopsoas; RF: rectus femoris; MH: medial hamstrings; TA: tibialis anterior; FLEXORS: EMG amplitude averaged across IL, RF, MH and TA; SOL: soleus; RMS: root mean square; JPD: joint probability density distribution; Co-co-Low: co-contraction at lower level of activation; Co-co-Hi: co-contraction at higher level of activation.
